# Impact of cataracts and cataract surgery on quality of vision: Impacto da catarata e da cirurgia de catarata na qualidade da visão

**DOI:** 10.1590/1516-3180.2015.01520108

**Published:** 2015-11-13

**Authors:** Eirini Skiadaresi, Colm McAlinden

**Affiliations:** I MD, MSc, FEBO. Ophthalmologist, Department of Ophthalmology, Singleton Hospital, ABM University Health Board, Swansea, United Kingdom.; II MB BCh, BSc (Hons), MSc, PhD. Doctor, Department of Surgery, Morriston Hospital, ABM University Health Board, Swansea, United Kingdom.

We read with interest the letter to the editor by Fazzi and colleagues who report a negative correlation between anxiety (measured with the IDATE) and quality of life (measured with the WHO-QOL-BREF) in 52 patients with cataracts.^1^ They state: "*This finding corroborates previous studies that have demonstrated low quality-of-life scores among patients with cataracts* 2..."

We would like to clarify that the cited paper did not assess quality of life in patients with cataracts and hence did not demonstrate low quality-of-life scores among patients with cataracts.^2^ The study assessed a different latent trait - quality of vision, using the Rasch-scaled Quality of Vision (QoV) questionnaire.^3^ ^4^ The QoV was completed before and three months after cataract surgery in 212 patients across four groups: first or second eye surgery, with or without ocular comorbidity. The study found that cataracts in one or both eyes causes a similar loss in subjective quality of vision, which is also irrespective of the presence of ocular comorbidity. Cataract surgery resulted in a large and comparable improvement in subjective quality of vision, regardless of ocular comorbidity and first or second eye surgery. 2 These improvements in quality of vision are in line with improvements using visual function and quality of life questionnaires following cataract surgery, which may indicate a relationship between these latent traits.^5^ ^6^
